# Online Assessment and Game-Based Development of Inductive Reasoning

**DOI:** 10.3390/jintelligence10030059

**Published:** 2022-08-18

**Authors:** Attila Pásztor, Andrea Magyar, Anita Pásztor-Kovács, Attila Rausch

**Affiliations:** 1 Institute of Education, University of Szeged, 6722 Szeged, Hungary; 2 MTA—SZTE Research Group on the Development of Competencies, 6722 Szeged, Hungary; 3 MTA—SZTE Digital Learning Technologies Research Group, 6722 Szeged, Hungary; 4 Institute of Education, Eötvös Loránd University, 1075 Budapest, Hungary

**Keywords:** inductive reasoning, assessment, game-based learning, Karl Josef Klauer’s model

## Abstract

The aims of the study were (1) to develop a domain-general computer-based assessment tool for inductive reasoning and to empirically test the theoretical models of Klauer and Christou and Papageorgiou; and (2) to develop an online game to foster inductive reasoning through mathematical content and to investigate its effectiveness. The sample was drawn from fifth-grade students for the assessment (N = 267) along with the intervention study (N = 122). The online figurative test consisted of 54 items: nine items were developed for each of the six inductive reasoning processes. The digital game-based training program included 120 learning tasks embedded in mathematical content with differential feedback and instructional support. The test had good psychometric properties regarding reliabilities, means, and standard deviations. Confirmatory factor analyses revealed that the six processes of inductive reasoning and the three latent factors of Similarity, Dissimilarity, and Integration could be empirically confirmed. The training program was effective in general (corrected effect size = .38); however, the process of cross-classification was not developed significantly. Findings could contribute to a more detailed understanding of the structure and the modifiability of inductive reasoning processes and could reveal further insights into the nature of fluid intelligence.

## 1. Introduction

Inductive reasoning is one of our fundamental thinking processes. It plays a central role in knowledge acquisition, in the transfer of knowledge and it is also strongly related to the development of higher-order thinking skills, to scientific reasoning and general intelligence (Carroll 1993; Demetriou et al. 2018; Dunbar and Fugelsang 2005; Klauer and Phye 2008; Molnár et al. 2013). Due to the rapidly changing economic and social environment, the importance of teaching general cognitive abilities such as inductive reasoning is constantly increasing (Mousa and Molnár 2020; Perret 2015; Tomic and Klauer 1996). Therefore, developing methods for enhancing reasoning skills in the classroom context and embedding them in curricula more explicitly have received growing interest over the last decades (Adey et al. 2007; Csapó 1999; Nisbet 1993; Resnick and Klopfer 1989). In order to develop cognitive abilities efficiently, detailed knowledge about the structure, development, and modifiability of the abilities is needed to guide the fields of classroom instruction and curriculum development (Christou and Papageorgiou 2007; Csapó 1999; Guerin et al. 2021). In addition, to support everyday educational practice, easy-to-use instruments and training programs are necessary to carry out assessments on a regular basis to monitor the development of the students and to foster reasoning skills (Csapó et al. 2012b). However, due to the limitations of traditional paper-based and face-to-face methods, regular assessment and systematic development of reasoning skills in an everyday classroom environment are hard to realize. A further major challenge is to find efficient ways to handle individual differences, thus fitting the instructional support to each student’s actual performance in a learning situation. The advantages of computer-based assessment and digital game-based learning such as innovative item design, immediate feedback, the possibility of handling individual differences, or creating playful learning environments could enable us to increase the efficiency of measuring and fostering cognitive abilities in an educational context (Csapó et al. 2012a, 2012b). 

Addressing these challenges, in our study, we aimed to investigate the structure and modifiability of inductive reasoning. To this end, based on a well-established model of inductive reasoning (Klauer 1990) we developed an online instrument using figurative items for assessing and a game-based intervention program embedded in mathematical content for fostering inductive reasoning in classroom settings.

### 1.1. Definition and Assessment of Inductive Reasoning

The nature and processes of induction have been discussed with respect to different philosophical and psychological traditions such as cognitive psychology or psychometrics (Carroll 1993; Heit 2007). In a general sense, induction is the process of finding meaningful patterns and formulating rules and hypotheses based on particular observations, and then generalizing these rules to unobserved cases. Thus, induction makes us able to infer the unobserved, to formulate novel conclusions and hypotheses about the unknown, and to generate new knowledge (Csapó 1997; Heit 2007; Sloman and Lagnado 2005). Induction moves from the particular to the general and it is probabilistic by its nature; therefore, the derived conclusions are only true with a certain degree of probability (Perret 2015). Inductive reasoning is often discussed and defined in comparison to deductive reasoning as the other main form of reasoning. The major distinctions are that deduction moves from the general to the particular and–if the premises are true–the conclusion must be true as well. However, there is still an ongoing discussion on whether the two types of reasoning are distinct from each other at the fundamental level (Goel and Dolan 2004; Hayes et al. 2018; Heit 2007; Stephens et al. 2018). 

In the psychometric tradition, inductive reasoning has been identified as one of the key factors of general intelligence (Carroll 1993). Spearman argued that the g factor is most influenced by inductive reasoning processes, as he referred to as the “education of relations” (Spearman 1923; cited by Klauer and Phye 2008). Inductive reasoning is usually operationalized and assessed by analogies, classifications, series completion problems, and matrices tasks (e.g., the Raven’s progressive matrices test or the Cattell Culture Fair Intelligence Test). Later factor analytic studies provided further empirical support for its central role in the structure of intelligence and in human cognition in general and showed the significance of inductive reasoning in fluid intelligence (Carroll 1993; Kovacs and Conway 2016; Sternberg 2018). Some researchers even refer to inductive reasoning as the “heart of general intelligence” (Adey et al. 2007, p. 80). 

Klauer’s view of inductive reasoning can be considered one of the well-structured and detailed theories in the field (Klauer 1990, 1996, 1999; Klauer and Phye 2008; Perret 2015). Klauer (1999) argued that a distinction should be made between inductive reasoning and inductive inference. In his view, inductive reasoning is the process when one detects regularities or rules based on a particular observation. Inductive inference goes beyond the scope of the situation, and an inference is made to the unobserved cases as well. Klauer provided an example to make the difference more obvious between the two processes: let us assume that a set of toys is given to a child. He/she recognizes that all of them are made of wood, this process could be referred to as inductive reasoning. He/she also makes a (false) conclusion that all toys are made of wood, this process corresponds to inductive inference. According to Klauer, the core process of inductive reasoning is comparison. During the comparison process, one could detect similarities and dissimilarities between the attributes and relations of the examined objects. Thus, Klauer defined inductive reasoning as detecting regularities and irregularities by finding out

A: {a1: similarity; a2: difference; a3: similarity and difference}
of
B: {b1: attributes; b2: relations}
with
C: {c1: verbal; c2: pictorial; c3: geometrical; c4: numerical; c5: other}
material (Klauer and Phye 2008, p. 87).

Facets A and B are representing the central parts of the definition. If we take the Cartesian product of these two sets, six processes of inductive reasoning can be formulated (Table 1). For example, in the case if Generalization similarity of attributes should be detected, the case of Recognizing relationships similarity of relations should be identified. Cross classification and System construction are representing processes in which both similarities and differences should be taken into account regarding attributes and relations respectively. Klauer also listed possible item formats for the different processes. For instance, Similarity of attributes can be assessed by class formation problems, Recognizing relationships with analogy or System construction with matrices tasks (Table 1). 

Despite its comprehensive nature, there is a lack of research on examining the construct validity of the model and testing whether the different processes can be empirically distinguished. de Koning et al. (2003) developed an instrument using item formats with real-life and geometric objects. A study was carried out among third graders, and the psychometric properties of the test were examined. The test proved to be reliable and valid; however, their results supported that the construct is unidimensional (de Koning et al. 2003).

**Figure 1:**
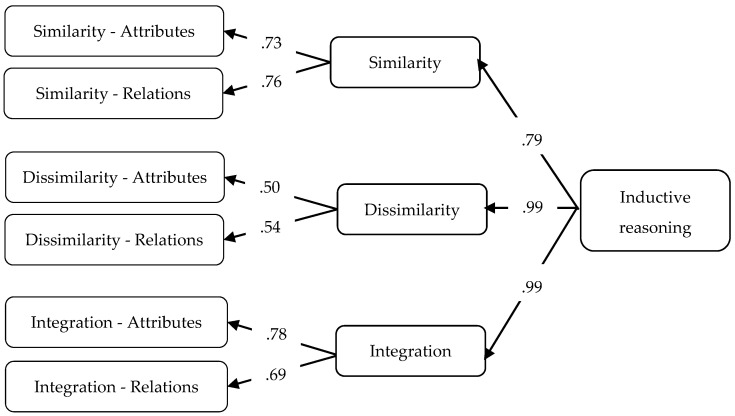
Hiearchical model with data parameters (factor loadings) suggested by Christou and Papageorgiou (2007, p. 63).

Christou and Papageorgiou (2007) also developed an instrument but using mathematical content (i.e., numbers) and conducted a study among five graders. They suggest that three key cognitive processes should be identified based on Klauer’s model: processes dealing with similarities of attributes and relations (Similarity: Generalisation, Recognizing relationships), processes dealing with dissimilarities (Dissimilarity: Discrimination, Differentiating relationships) and Integration where both similarities and dissimilarities have to be taken into account to solve a particular task (Cross classification, System construction). In their model, they included the six processes as a first order factor, they defined the three key cognitive processes as a second order factor, and a third order factor was also formulated representing inductive reasoning as a general latent construct. Figure 1 shows the model visually with the factor loadings. The proposed model was consistent with the data; they reported good fit indexes (Christou and Papageorgiou 2007). Thus, the results suggested that the six processes and the three key cognitive processes could be empirically distinguished. However, so far no research confirmed the construct validity of the model in the case of applying domain general stimuli such as items with figurative content. 

### 1.2. Fostering Inductive Reasoning in Educational Settings

Based on the theoretical model Klauer developed training programs in order to foster inductive reasoning in various age cohorts (Klauer 1989, 1991, 1993). Program I. consisted of pictorial and manipulative tasks, and it was applied among 5–8 years old children (Klauer 1989). Program II. targeted 11–13 years old students, and it devised with textual and numerical learning tasks (Klauer 1991). In program III. pictorial, numerical, and textual tasks were applied for enhancing students’ thinking skills with learning disabilities aged 14–16 years (Klauer 1993). All programs consisted of 120 learning tasks which means that there were 20 tasks for each inductive reasoning process. The content of the programs was not based on a specific school subject; they applied various contents such as problems and materials relating to daily life. However, as learning at school is also part of everyday experiences for older students, a portion of the learning tasks were related to subject materials as well (Klauer and Phye 2008). The trainings were paper-based, and they were conducted in face-to-face situations where the teachers provided guided instructions for the students during the training (see for example in de Koning and Hamers 1999). The implementation of these programs lasted from 2 to 5 weeks.

Numerous studies demonstrated the effectiveness of these trainings in different contexts such as in various cultures, ages, in different target groups (average or gifted students, students with special needs), or in different settings (training individually, in pairs, or in class) (Barkl et al. 2012; de Koning and Hamers 1999; de Koning et al. 2002; Hamers et al. 1998; Klauer 1996; Klauer and Phye 2008; Klauer et al. 2002; Tomic 1995; Tomic and Kingma 1998; Tomic and Klauer 1996). These studies generally used traditional fluid intelligence tests with figurative items such as the Raven progressive matrices or the Cattel Culture Fair test to check the effectiveness of the treatment. In a comprehensive meta-analysis, Klauer and Phye (2008) reported that the average effect size of the programs was d = .52 (range = 0–1.25). As in some cases achievements of the control group also increased, and a corrected effect size was used. This means that the effect size of the control group was subtracted from the effect size of the experimental group. Further transfer effects were examined by experimental situations where children participated in a learning session. Students were taught a lesson in an academic subject that had not been taught before (e.g., mathematics, physics, biology, grammar, reading comprehension). An informal criterion-referenced test was administered before and after the lesson. Thus, they examined the effect of the training on academic learning. Results showed that the corrected effect size was even larger (d = .69; range = .13–1.63) than the effect on intelligence measures although the latter case represented a smaller transfer distance.

### 1.3. Possibilities of Technology-Based Assessment and Game-Based Learning in Fostering Inductive Reasoning

The Cognitive training for children was originally developed as a paper-based program, and it was used in a face-to-face condition. Implementing these kinds of programs is time-consuming and expects significant extra workload from the classroom teachers. Administering the data collections for the pre- and posttests, analyzing data, and carrying out the experimental sessions, especially at the individual level, is time demanding. Thus, the possible integration of these programs in everyday classroom practice or at the system level is limited.

Technology may provide solutions for overcoming these limitations. Administering the tests via technology can significantly reduce the time and costs of the testing process. Automatic scoring and evaluation, and the possibility of providing immediate feedback are attributes that can lead to the development of more efficient, timely and cost-effective, easy-to-use instruments (Csapó et al. 2012b). Besides testing, technology could also offer solutions for designing other forms of learning opportunities (Csapó et al. 2012a). For example, applying digital games to education is a rapidly developing research field (Chen et al. 2020). The question is not whether digital game-based applications can be effective or not, but rather how these tools should be designed to harness their learning potential, and how they could be integrated into educational practice efficiently (Kucher 2021; Wouters et al. 2013). 

For instance, Kucher (2021) identified five key principles that should be considered to facilitate the effectiveness of digital game-based learning: interactivity, immersiveness, adaptive problem solving, feedback, and freedom of exploration. Personalized instructional support, motivating learning environment, and embedding different learning tracks based on the actual performance are also highlighted as important factors in game design to increase effectiveness (Cai et al. 2022; Csapó et al. 2012a; Wouters and van Oostendorp 2013). In addition, game-based learning also allows us to train students in larger groups without the need for a permanent teacher presence.

Appling these advantages some research has been conducted to adapt Klauer’s training program to a computer-based environment. In a modified paper-based version of the original program (Molnár 2011), the learning tasks were migrated to a computer-based environment (Csapó et al. 2012a; Molnár et al. 2012). The program proved to be effective among first and second-grade students, and there were no significant differences between the face-to-face and the game-based condition (Molnár and Lőrincz 2012). In Pásztor’s online training program (Pásztor 2014a, 2014b, 2016), a content-based approach was implemented (Csapó 1999), thus the learning tasks were embedded in mathematical content. Students received immediate feedback after every learning task and in case of failure, instructional support was provided to guide the learning process. Although the program could be considered playful, no additional game elements such as background story or defining a specific goal for the game were integrated. The effectiveness of the program was investigated among three- and fourth-grade students. A computer-based test was developed based on Klauer’s model and applied as a pre- and post-test. Although the reliability of the whole test was sufficient (Cronbach’s alpha = .83), the subtests were less reliable (values ranged from .38 to .67). The corrected effect size of the program was d = .33, indicating the effectiveness of the training in general; however, in the case of Discrimination and Cross classification the developmental effect was not significant. This version of the program was translated into Arabic and tested among fourth- and fifth-grade students (Mousa and Molnár 2020). The effectiveness of the program was measured by figural and number series and analogies, and it proved to be effective (Cohen’s d = 1.71).

### 1.4. The Present Research

The aim of the research is twofold. The first objective is to develop a domain-general online assessment tool for inductive reasoning based on Klauer’s model and to analyze its psychometric features and to empirically test the theoretical models suggested by Klauer (1990) and Christou and Papageorgiou (2007). The second aim is to further develop Pásztor’s computerized learning program and to test its effectiveness among five graders. To this end, our research questions are the following:

RQ 1: What are the psychometric features of the online figurative test?
RQ 2: Is Klauer’s model empirically supported by our data?
RQ 3: Is the hierarchical model suggested by Christou and Papageorgiou empirically supported by our data?
RQ 4: Does the training program effectively develop inductive reasoning in grade 5?
RQ 5: How does our intervention program affect the development of the different inductive reasoning processes?

## 2. Materials and Methods

### 2.1. Participants

The samples of the studies were drawn from grade 5 students. As the assessment and the intervention studies were conducted in different calendar years, separate sampling procedure was carried out for the two studies. In the study examining the psychometric features of the online figurative test, 267 pupils participated (age mean = 11.1 years, SD = .42 years; male proportion: 44.9%) from five schools, altogether from 11 classes. In the intervention study, three schools were involved with altogether 141 students. 19 students were left out from the analyses due to the missing pre- or posttest measure, thus 122 pupils formed the basis of the final analyses (age mean = 11.2 years, SD = .43 years; male proportion: 43.4%). Three classes of one school represented the experimental group, N = 67, and three classes from the other two schools formed the control group, N = 55.

### 2.2. Instruments

#### 2.2.1. The Online Assessment Tool

**Figure 2:**
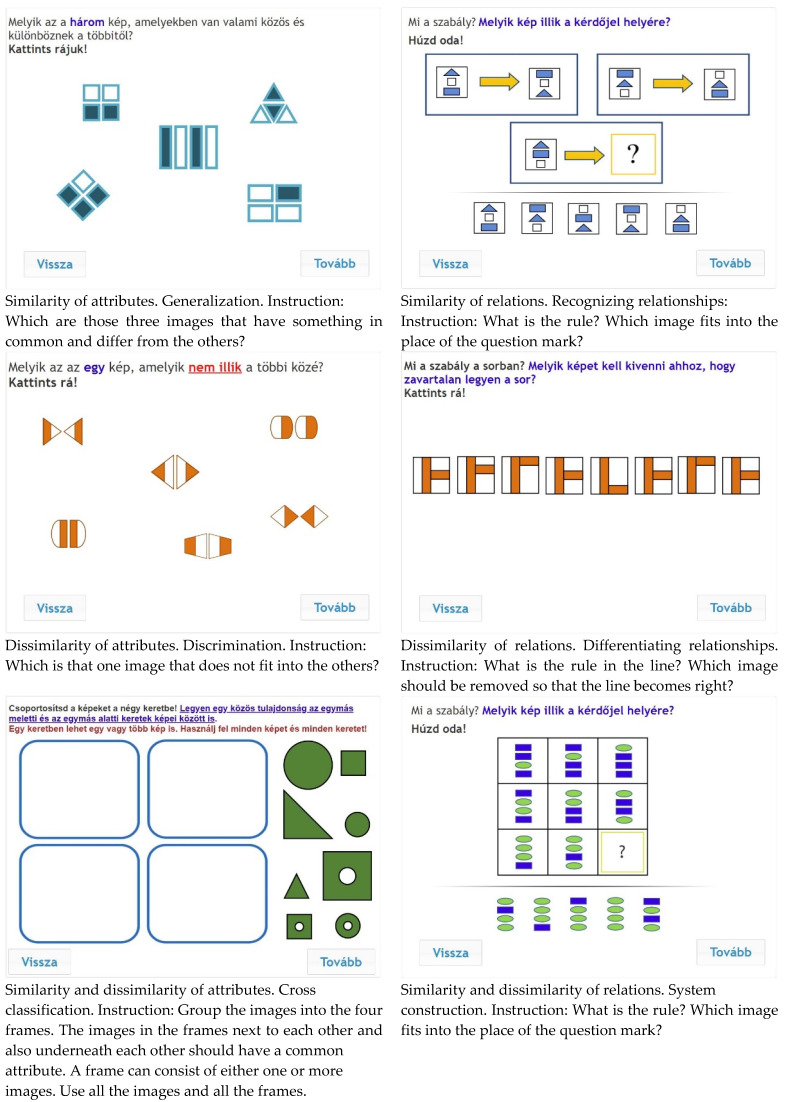
Sample items from the inductive reasoning test.

The online figurative test consisted of 54 items: nine items were developed for each scale described by Klauer’s theory. Figure 2 shows an example for each item type. Generalization was operationalized as class formation: students had to find three images out of five which had something in common. Discrimination was measured by identifying the disturbing item in a set (odd one out task). In Cross classification tasks, students had to classify eight objects into four sets (4-fold scheme). Recognizing relationships items were operationalized as figural analogies: two examples were provided, and students had to complete the third analogy. Differentiating relationships were assessed with disturbed series, and finally, matrices tasks were used for System construction. All items were scored dichotomously. During the test development process, 5 modified items were adapted from Pásztor’s (2016) instrument. 

#### 2.2.2. The Online Training Program: Save the Tree of Life 

**Figure 3:**
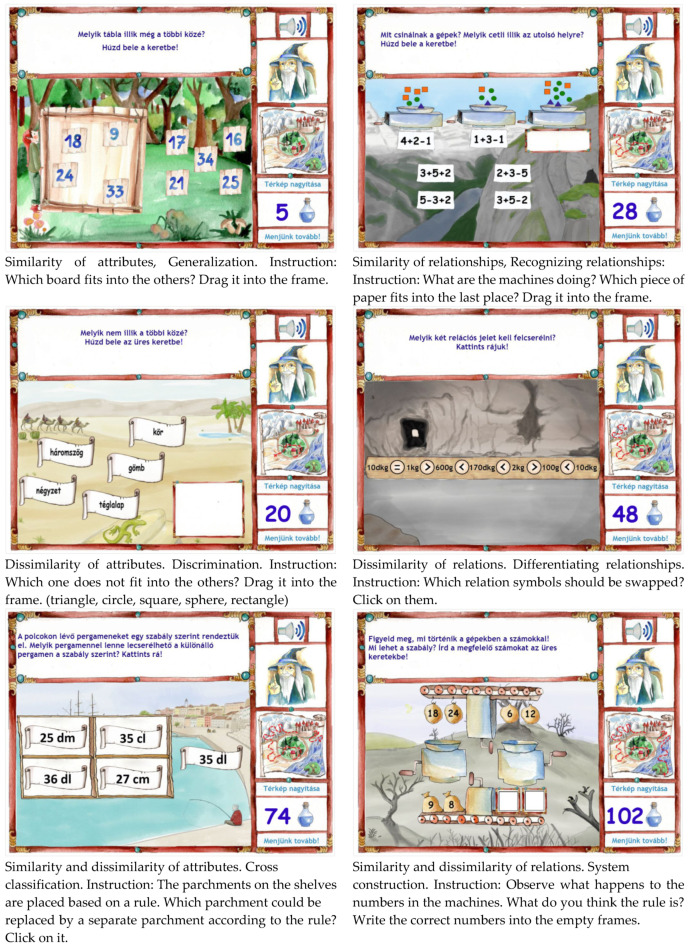
Sample learning tasks from the training program.

The online training program was also based on Klauer’s model of inductive reasoning and on his concept of Cognitive training for children (Klauer 1989). It consisted of 120 learning tasks, 20 for each inductive reasoning process, embedded in mathematical content. Figure 3 shows examples from the training program for each inductive reasoning process. The development of the training was based on Pásztor’s (2014a, 2014b, 2016) program but major modifications were implemented: the learning tasks were reconsidered both in terms of the content and operations, the content of instructional support, and the feedback mechanism changed radically, the order of the learning tasks were also modified, and the tasks were embedded in a game-based environment. 

The content of the learning tasks was based on third- and fourth-grade mathematics textbooks and workbooks. A variety of mathematical topics were covered including basic operations, the relationship between numbers and quantities, the use of relational symbols, even and odd numbers, Roman numerals, geometric concepts and transformations, measurements, unit conversions, sequences (continuation, ordering), data pairs, relationships between sets of data and measuring time. 

Besides the item types presented for the assessment instrument, further task schemes were also applied to increase the variability of the activities. For example, for Generalization class formation and class expansion tasks were also used, in some differentiating relationship tasks, students had to find those elements which had to be swapped or in cross classification tasks, the four sets were already filled, and students had to find the correct settings for a given element (see Figure 3). Furthermore, reducing the trial-and-error strategy we tried to develop as many open-ended training tasks as possible. For example, in series completion tasks, students had to type the numbers instead of choosing one from four alternatives. 

To increase students’ motivation and to help them overcome any possible aversion to mathematics we have embedded the training tasks into a game-based environment. A simple backstory was developed using common archetypes (e.g., wise old man, Jung 1968): the Tree of Life is threatened by extinction, thus a hero is needed to prevent this catastrophic event by collecting as many potions of water of life as possible. The Wise Old Man knows where these potions could be found but the only way to obtain them is to unlock the spells they are protected by, which requires them to correctly solve the training tasks. The Wise Old Man also guides the player through the game by providing encouragement and instructional support in the tasks. During the adventure, the player must visit different regions of the empire (see the map on the right side of the screenshots in Figure 3) and finally must water the Tree of Life. On the bottom right of the screen, students could monitor the number of collected potions of water of life (see Figure 3).

To increase learning effects feedback loops were developed for each of the 120 tasks. In the initial condition, the students could solve the tasks without any additional support (see the examples in Figure 3). Once an answer was given and the students clicked on the ‘Let’s move on button!’, the system provided immediate feedback. In case of an incorrect answer, learners received constructive feedback which was always formulated to encourage the application of the inductive reasoning process required to solve the task. For instance, in the case of the recognizing relationships task in Figure 3 (analogy task), the system gave the following instructional support: ‘Think again! What is the relationship between the colored shapes and the numbers on the tags?’ Or in case of the differentiating relations task: ‘Let’s rethink it! What could be the rule? What relations could you differentiate between the objects?’.

**Figure 4:**
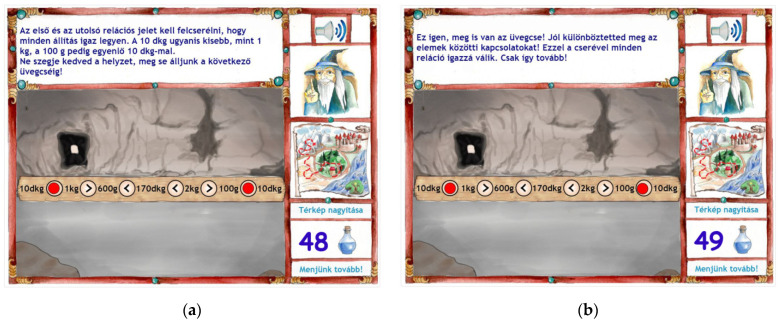
Samples for feedback in the training program at the end of the feedback loop. (**a**) In case of a third unsuccessful trial of the learning task, instruction: The first and the last relation symbols should have been swapped to make all the statements true. Namely, 10 dg is smaller than 1 kg, and 100 g is equal to 10 dg. Don’t be discouraged; let’s get the next potion of water of life. (**b**) In case of the correct solution of the task: Well done, you’ll get the bottle. You have differentiated the relations between the objects correctly. With this swap all the statements are true. Keep it up.

Besides this instructional support, a Help button popped up. If students clicked on it, further guidance was provided in connection with the mathematical content of the task. For the recognizing relationships task mentioned before, the guidance was the following (Figure 3, analogy task): ‘Examine which shapes could represent which number. Pay attention to the operations as well.’ For the differentiating relations task (Figure 3, disturb series), this message appeared: ‘Try to express the quantities in the same unit and examine the relational symbols between them. Are all the statements true? Here is some help for the conversion of the units: 1 dg = 10 g, 10 dg = 100 g, 1 g = 100 dg = 1000 g, 1 t = 1000 kg).’ If the students gave a wrong answer again, a similar but rephrased instructional support was provided, and they could retry to solve the task. The content of the Help button remained the same. In case of a third unsuccessful trial, the software showed the solution with an explanation of how the task should have been solved (Figure 4a). The intent behind this design was to reduce the frustration of the students and to facilitate the learning process and understanding. If the correct solution was provided, students received positive, reinforcing feedback. This feedback also gave an indication of the applied thinking process with the aim of strengthening the learners’ metacognitive awareness (Figure 4b). 

To sum up, the developmental impact of the program is twofold: on the one hand, it provides opportunities to practice different mathematical operations and thus deepen mathematical knowledge. On the other hand, with the application of the instructional support messages, it encourages students to identify and differentiate attributes and relationships between the components in the task to enhance the use of the inductive processes according to Klauer’s model.

### 2.3. Procedures

The assessments were carried out through the eDia system (Csapó and Molnár 2019; Molnár and Csapó 2019). Teachers received a description of the test with a general overview of Klauer’s model of inductive reasoning. An assessment guide was also provided to ensure the standardized process of the data collection. The test was administered in the schools’ computer labs using desktop computers. Students could log into the system with anonymous identifiers. After the test completion, students received immediate feedback on their overall performance. Teachers could download feedback related to the six different inductive processes from the system. Each testing procedure was carried out within a 45-min lesson, the average testing time was 26.6 min (SD = 7.0 min). 

The training program was available through the eLea platform (Molnár et al. 2019). Teachers received the test description, the assessment guide, and an overview of the training program. To examine the effectiveness of the intervention program the same test was applied in the intervention study as in the assessment study. With this experimental design, the near transfer effects of the training could be avoided as the test developed with general (i.e., figurative items) and the intervention was embedded in mathematical content.

Students used their own anonymous identifiers to log into the pre- and post-test and to the training program. One week after the pretest, the students in the control group continued their regular instruction while the experimental group participated in the online training which lasted for five weeks. One week after the last session, the posttest was administered. In each training session, the students went through 24 learning tasks during a 45-min lesson. We distributed easy and difficult learning tasks for each session but there were somewhat more challenging tasks in the later sessions.

Teachers were instructed to give no additional instructional support to the students during the training; they only supervised the sessions and were allowed to provide help if there were any technical difficulties. The game provided all the necessary information to go through the training including the understanding of the backstory, the operations of the buttons, and the game mechanics. Students could listen to all instructions via headphones. All the data collections were carried out in the computer labs of the schools. 

## 3. Results

### 3.1. Assessment of Inductive Reasoning 

The reliability of the whole test was high, Cronbach’s alpha = .91. The subtests also had acceptable reliability indices (Table 2). The values for Discrimination and Differentiating relationships were somewhat lower-below .70-but considering the number of the items they still could be in the acceptable range. The item-total correlation analyses showed that all items positively correlated with the total test score. No significant improvement could be reached connected to reliability with item deletion; therefore, all items were kept for further analyses. 

Confirmatory Factor Analysis (CFA) was carried out to examine the construct validity and the underlying model for inductive reasoning. Theta parameterization and WLSMV estimator were applied in the analyses. The results showed that the 6-dimensional model defined by Klauer’s theory fitted well to the data (Table 3), and the Chi-squared difference tests showed that it fitted significantly better than the 1-dimensional model (χ^2^ = 2317.05; *p* < .01). Thus, the six latent factors of inductive reasoning were empirically distinguished (Table 3). 

**Figure 5:**
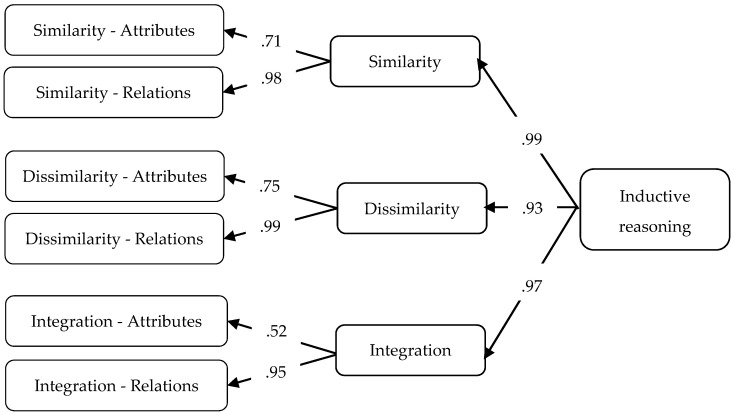
Results of the hierarchical model (Christou and Papageorgiou 2007, p. 63) with factor loadings.

The model fit for the hierarchical model suggested by Christou and Papageorgiou was also good: χ^2^ = 1615.58 df = 1371 *p* <.01, CFI = .942, TLI = .940, RMSEA = .026 (95% CI: .020–.031). The loadings of the second-level latent factors were high in general, however, the value for Cross classification (Integration–Attributes) was somewhat lower (Figure 5). A tendency could be noticed that loadings regarding the factors dealing with relations were higher. The loadings on the third order level were high as well, providing further support that the hierarchical model is consistent with the theory. In general, the model proved to be consistent with the data indicating that besides the six processes of inductive reasoning the three latent factors of Similarity, Dissimilarity and Integration could also be empirically demonstrated.

To further analyze the construct validity of the instrument, correlation analyses on the manifest level were also conducted. Table 4 shows the correlation coefficients of the Inductive reasoning test and its subtests. All subtests were strongly correlated with the whole test, the values ranged from .59 to .84 indicating that all processes were playing an important role in inductive reasoning. The moderate correlations between the subtests provide further empirical support that all processes represent distinguished dimensions of inductive reasoning. The magnitudes of the coefficients were the lowest in the case of the Cross classification, the values ranged between .29 to .38, which implies that this process fits less into the overall picture of the construct. The correlation coefficients between Similarity and Dissimilarity (r = .70) and between Integration (r = .64 and .63 respectively) were high but the values also indicate that the three key cognitive processes are distinct from each other.

The overall mean achievement was 48.01%, thus the difficulty of the test fitted well with the students’ skill level (Table 5). Most mean scores of the subtests were also close to the psychometrically ideal 50%. The achievements in Cross classification and Differentiating relationships were somewhat lower (37.04% and 39.58% respectively) but they are still in an acceptable range. It seems that these tasks were more difficult for the students in the sample. The standard deviations ranged between 18.67–28.33% which indicates that the test and all its subtests sufficiently differentiate between low and high skills students (Table 5).

### 3.2. Fostering Inductive Reasoning

The reliability of the whole test was high for the pre- and the posttest as well (in both cases Cronbach’s alpha = .90). The values for the subtests showed similar patterns to the assessment study. In some cases, the reliability increased but there were also some decreases. The lowest value was .55 for Differentiating relationships in the pretest; however, the same subtest in the posttest had a .65 alpha value which was higher than in the assessment study (.63). In general, the assessment tool worked appropriately in the experimental study as well. 

There was no significant difference in the achievements between the control and the experimental group in the pretest (Table 6). The scores for both the control and the experimental group significantly increased from the pretest to the posttest. However, the development of the experimental group was significantly greater than the control group’s achievement (Table 6). Based on the development of the experimental group the effect size of the training was Cohen’s d = .63. As the control group also significantly improved (Cohen’s d = .25) the corrected effect size was calculated as well. This procedure resulted in the corrected effect size of .38. The average number of the collected water of life potions in the sessions were 19, 19, 17, 16, and 16 respectively. The maximum number of potions was 24 for each session; therefore, this result suggests that the students were immersed in the game in general. 

**Figure 6:**
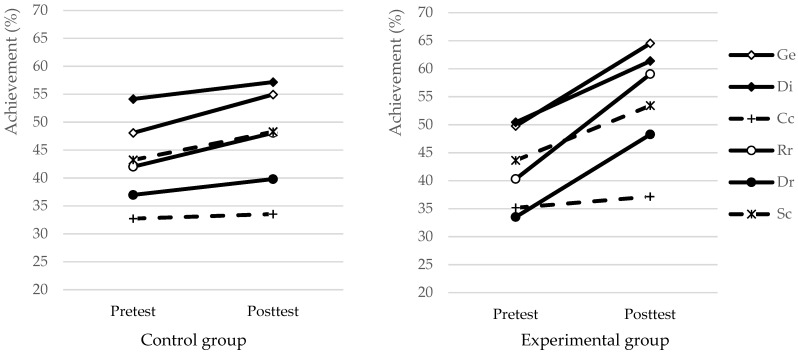
Changes in performance regarding the inductive reasoning processes for the control and the experimental group. Note: Ge: Generalization, Di: Discrimination, Cc: Cross classification, Rr: Recognizing relationships, Dr: Differentiating relationships, Sc: System construction.

Table 7 summarizes, and Figure 6 visually depicts the changes in performance regarding the different inductive reasoning processes. In case of the control group, there was no significant development except in Generalization. For the experimental group, except for Cross Classification, all inductive reasoning processes developed significantly. Based on the corrected effect sizes, the largest developmental effects occurred in Recognizing and Differentiating relationships. In the latter case, the effect reached .5 of the standard deviation. Similar effect sizes in magnitude were found for Generalization, and Discrimination, students’ performance increased by one-third standard deviation in both processes. Table 7 also shows the changes in the three main factors of inductive reasoning. In case of Similarity, both groups developed significantly during the period of the training while for the two other factors only the achievements of the experimental group increased significantly. For Similarity and Dissimilarity, the corrected effect sizes are similar in magnitude, close to 0.5 standard deviations. The developmental effect is much smaller for Integration, the corrected effect size is .13 (Table 7).

## 4. Discussion

Inductive reasoning plays a fundamental role in organizing and applying knowledge and strongly relates to general intelligence. Thus, its relevance in an educational context is well-grounded for theory and practice. However, due to the limitations of traditional paper-based and face-to-face methods, it is hard to realize its systematic enhancement in everyday school practice. Based on Klauer’s well-established model and his widely tested paper-based intervention program, we aimed to develop easy-to-use online instruments for assessing and fostering inductive reasoning. Only a few studies were carried out to examine the construct validity of the model and to investigate the possibilities of embedding the program in school subjects. Addressing these research gaps, we also tested the dimensionality of the theoretical model, and we empirically examined the modifiability of these dimensions through an online training embedded in mathematical content among five-grade students.

### 4.1. Assessment of Inductive Reasoning Strategies

The psychometric properties of the online test were good in general. The reliability of the whole test was high, although Cronbach’s alpha values for the Discrimination and the Differentiating relationships were somewhat lower. The analyses of means and standard deviations showed that the difficulty of the test was suitable for the age cohort in the study, and the test could differentiate between students with low and high performances.

Klauer’s (1990) model of inductive reasoning proved to be consistent with our data providing empirical evidence for distinguishing the six inductive processes. Although the 6-dimensional model fit was significantly better, the fit indices for the 1-dimensional model were also acceptable. This result also fits the theory and could explain the unidimensional nature of the construct found in the study of de Koning et al. (2003) as it mirrors the core comparison process of inductive reasoning suggested by Klauer.

The hierarchical structure of inductive reasoning suggested by Christou and Papageorgiou (2007) was also supported by our data. The three key cognitive processes, namely Similarity, Dissimilarity, and Integration were empirically demonstrated. As we used figural items as opposed to the mathematical content of Christou and Papageorgiou’s test, this result represents further evidence for the validity of these three key cognitive processes. The factor loadings for the six inductive reasoning processes were high, although a tendency could be observed that the loadings for processes dealing with attributes are somewhat lower, especially in the case of Cross Classification. This pattern also can be recognized in the results of Christou and Papageorgiou (2007), which may indicate that dealing with relationships represents more influential factors in inductive reasoning. This assumption is also supported by previous findings in which the significant role of analogies (Recognizing relationships in Klauer’s model) was argued in the processes of inductive reasoning (Csapó 1997; Pásztor 2016; Pellegrino and Glaser 1982; Sternberg and Gardner 1983). The correlation analyses on the manifest level provided further support for construct validity; the magnitudes of the coefficients were consistent with the theory. The correlations between the subtests were moderately strong, implying that the six processes positively relate to each other. However, this result also indicates that it is worth measuring them separately as they all represent different aspects of inductive reasoning. In line with the findings from factor analysis, Cross classification–the process of dealing with similarities and differences of attributes simultaneously–showed moderate fit to the construct.

To summarize our findings regarding the assessment of inductive reasoning (RQ 1–3), the test is suitable for assessing students’ inductive reasoning, and provides information about the developmental level of the different inductive reasoning processes. As the format of the instrument is close to conventional measures of fluid intelligence it could give a more differentiated picture—a student’s skill profile—in some degree of fluid intelligence as well. 

### 4.2. Fostering Inductive Reasoning Strategies

The corrected effect size of the training program was moderate, d = .38, which fits the general picture of previous research findings as the average effects of the training on intelligence was d = .52 (Klauer and Phye 2008). As our newly developed test can show the development of all the six processes, we could examine the effects in more detail. 

The results showed that all processes developed except Cross classification. This finding is in line with results from the assessment study as we found that Cross classification is less fit to the construct of inductive reasoning (i.e., weaker correlation coefficients and lower factor loadings). Thus, this result is another indication that this process behaves somewhat differently from the others. One possible reason would be the difference between test formats in the assessment tool and the training program. In the test items, students had to classify eight objects into four frames, while in the training tasks, they received an already filled 2 × 2 frame, and they had to decide which object in the frames could be substituted with the object next to the frame (see Figure 2 and Figure 3). However, in the case of other processes, we also used some other item formats in the training which were not applied in the test, and in these processes, there was a significant improvement. Furthermore, in Pásztor’s (2016) test version, the item formats were identical in the test and in the training (i.e., the already filled 2 × 2 frame type), but Cross classification did not improve significantly either in his experiment. Thus, different item formats between the test and the intervention don’t provide a satisfying explanation for the different nature of Cross classification. A reasonable assumption can be made that Cross classification could be the most challenging reasoning process to be fostered. At this point, it is worth noting that the second lowest effect size was in the case of System construction (d = .20). Both processes belong to the main process of Integration suggested by Christou and Papageorgiou (2007). Thus, it seems that detecting both similarities and differences simultaneously could be the processes that are challenging to develop. This assumption fits the theory as these integration processes represent a higher level of cognitive demand compared to the other processes. Another interesting tendency also can be detected in our results: the effect sizes for processes dealing with attributes are lower compared to the processes dealing with relations (see Table 7). Our data are not providing a solid basis for formulating a possible explanation for this phenomenon, but it highlights the importance of the attribute-relation aspect suggested by Klauer’s model. Thus, these findings are consistent with the theory from the perspective of the three key reasoning processes and from the perspective of the attribute-relation dimension as well. If fewer developmental effects could be expected in the case of dealing with attributes and applying the Integration processes, Cross classification should be the most challenging process to develop. 

However, some contradicting results could also be found in the literature as Klauer examined different transfer hypotheses as well (Klauer 1990). In two experiments, the intervention was carried out with figural content and the effect was measured by verbal and figural content. Regarding Cross classification, large effect sizes were reported even in the figural training–verbal test condition (d = .95 and 1.27). These experiments were conducted among educationally retarded children, the sample sizes were small (N = 10 and 12 respectively), and the intervention was conducted in a face-to-face context. Nevertheless, these results call attention to the importance of different factors influencing the developmental effect of the training. 

A further issue to discuss is related to the integration of the advantages of game-based learning into the program. The training has been radically further developed compared to the original Pásztor’s (2016) version in terms of the content of instructional supports, the feedback mechanism, and the appearance (e.g., visualization, background story). The corrected effect size of the original program was d = .33 so despite these upgrades the effect size only slightly increased (d = .38). At this point, it is hard to make far conclusions for several reasons. For instance, the age cohort was different in the two studies (3–4 graders and 5 graders respectively), and in both cases, the samples were not large. In addition, as the meta-analyses of Klauer and Phye (2008) showed, there is a large variance in the effect sizes among the different experimental studies (e.g., on intelligence the range was 0–1.25). A large variance has been detected in the case of this program as well: in the study testing the original version of the training in the Arabic context, the effect size was remarkably large (d = 1.71), although the test used for the pre- and post-test was different (Mousa and Molnár 2020). Nevertheless, our study also showed that effectively integrating digital game-based elements into educational programs is a challenging endeavor (Young et al. 2012). 

To sum up, our findings with regard to fostering inductive reasoning (RQ 4–5), our results have given further empirical support for the efficacy of Klauer’s training concept. The modifiability of the different inductive reasoning processes has been demonstrated and discussed. In addition, further evidence has been provided for effectively adapting the content-based method in Klauer’s approach and the possibility of migrating the training concept to a digital game-based environment (Csapó et al. 2012a; Mousa and Molnár 2020; Pásztor 2014a, 2014b, 2016).

### 4.3. Limitations and Further Research

While our study contributes to the field, it certainly has its limitations that also point out the possible directions for further research. The psychometric properties of the test were good in general; however, the analyses also revealed that two subscales (Discrimination, Differentiating relationships) need further development. The generalizability of our findings is limited as we only have data in grade 5, and especially in the intervention study, the sample size was rather small. Further research could be carried out in other age cohorts and larger samples so that we can learn more about the development and the nature of the processes and the usability of the test as well. Based on the psychometric properties of the instrument, it can be assumed that it could be used effectively from grade 4 to grade 6; however, empirical research should be conducted to confirm this hypothesis.

Our assumptions regarding the nature and modifiability of the cross-classification process, the three key cognitive processes, and the possible differences in the attribute-relation dimension should also be further examined. The modifiability could be affected by several factors such as the content of the learning tasks and the assessment items (i.e., transfer distance), the properties of the sample (e.g., age, motivation, cognitive abilities) or the mode of intervention (e.g., online, face-to-face, training in individual or in group conditions). These assumptions should be considered fruitful hypotheses for further studies. At this point, log file and distractor analyses can be conducted on the current data set to investigate these issues. 

Another important further research direction is to explore the source of individual differences in the inductive reasoning achievements and also in the extent of the developmental effects by administering background variables in future studies (e.g., socio-economical background, motivation, or students previous game experience and preferences for the game genre). As the intervention program was embedded in mathematical content, the developmental effects on mathematical knowledge also should be examined. In addition, assessing mathematical knowledge during the pre- and post-test would also allow to examine the effects of previous mathematical knowledge on the effectiveness of the program. Near transfer effects could also be explored using assessment items embedded in mathematical content. Transfer effects on academic learning can be investigated by applying the design of teaching a lesson in different academic subject areas and administering criterion-referenced tests after the learning session (Klauer and Phye 2008). In addition, longitudinal studies could explore the durability of the effects. In order to study whether the training effects can be unambiguously attributed to the inductive ingredients of the training program, further control groups should be included in the research design (i.e., children playing with another computer game with no inductive requirements).

### 4.4. Pedagogical Implications

Our study confirmed that the precise definition and structure of Klauer’s model make a solid basis for assessments and developmental purposes in educational settings. The findings can provide insights for teachers and curriculum designers into the integration of assessing and fostering students’ inductive reasoning in primary education more effectively.

Our research also showed that integrating Klauer’s concept of inductive reasoning into classroom teaching could be strengthened by applying the advantages of technology-based assessment and digital game-based learning. 

Measuring and fostering students’ skills with traditional methods is time-consuming and costly (e.g., test administration, data analysis), and because of the delayed feedback on students’ performance, the effective integration of the data in the classroom context is challenging. Moreover, carrying out interventions is even more demanding for the teachers, especially if the training is conducted at an individual level.

With the advantages of a computerized environment, our test can be administered more easily in a 45-min lesson, and it provides immediate feedback for both students and teachers on different dimensions of inductive reasoning. The online training program enables us to foster inductive reasoning in larger groups. The game-based environment and the background story could increase students’ engagement and motivation to learn. The program also gives immediate feedback, it guides the learning process with the application of the instructional support messages which are based on the students’ performance in each learning task. Thus, it considers individual differences to a certain degree, and it does this task in an automatized way. Using the eDia and eLea platforms, teachers could download more detailed feedback and monitor the students’ progress in the training as well. Thus, both the online assessment tool and the training can be considered easy-to-use instruments and be applied effectively in everyday educational practice.

## 5. Conclusions

Our study demonstrated an example of how to apply the advantages of technology to address the challenges in regular assessment and systematic development of thinking skills in a classroom context. According to our knowledge, no research was conducted to examine the construct validity of Klauer’s model applying only figural items and there is a lack of research investigating the modifiability and development of the six strategies defined by the model. The online training program could also be considered a pioneering enterprise in applying the advantages of the content-based method and digital game-based learning into Klauer’s training concept. Our findings could contribute to a more detailed understanding of the structure and the modifiability of inductive reasoning processes and could reveal further insights into the nature of fluid intelligence (Guerin et al. 2021).

## Figures and Tables

**Table 1:** Types of inductive reasoning problems (Klauer and Phye 2008, p. 88).

Process	Facet Identification	Cognitive Operation Required	Item Formats
Generalization	a_1_b_1_	Similarity of attributes	Class formation
			Class expansion
			Finding common attributes
Discrimination	a_2_b_2_	Discrimination of attributes	Identifying disturbing items
Cross classification	a_3_b_1_	Similarity and difference in attributes	4-fold scheme
			6-fold scheme
			9-fold scheme
Recognizing relationships	a_1_b_2_	Similarity of relationships	Series completion
			Ordered series
			Analogy
Differentiating relationships	a_2_b_2_	Differences in relationships	Disturbed series
System construction	a_3_b_2_	Similarity and difference in relationships	Matrices

**Table 2:** The reliability of the instrument and its subtests (Cronbach’s Alpha).

Subtests	Number of Items	Cronbach’s Alpha
Generalization	9	.77
Discrimination	9	.61
Cross classification	9	.71
Recognizing relationships	9	.76
Differentiating relationships	9	.63
System construction	9	.72
Inductive reasoning strategies	54	.91

**Table 3:** The goodness of fit indices for testing dimensionality of inductive reasoning strategies.

Model	χ^2^	df	*p*	CFI	TLI	RMSEA (95% CI)
1 dimension	1752.97	1377	.01	.911	.908	.032 (.027–.036)
6 dimensions	1454.23	1362	.04	.978	.977	.016 (.004–.023)

Note: df: degrees of freedom; CFI: Comparative Fit Index; TLI: Tucker–Lewis Index; RMSEA: Root Mean Square Error of Approximation; χ^2^ and df are estimated by WLSMV.

**Table 4:** Correlations among Inductive reasoning strategies test and its subtests.

Subtests	IND	Ge	Di	Cc	Rr	Dr
Generalization (Ge)	.73					
Discrimination (Di)	.72	.50				
Cross classification (Cc)	.59	.31	.29			
Recognizing relationships (Rr)	.84	.52	.51	.37		
Differentiating relationships (Dr)	.79	.48	.49	.38	.62	
System construction (Sc)	.80	.43	.48	.34	.70	.63

Note: All correlations are significant at the 0.01 level.

**Table 5:** Means and standard deviations of the instrument.

Subtests	Number of Items	Mean (SD) %
Generalization	9	54.02 (26.85)
Discrimination	9	57.01 (22.76)
Cross classification	9	37.04 (24.29)
Recognizing relationships	9	53.02 (28.33)
Differentiating relationships	9	39.58 (21.37)
System construction	9	47.40 (26.10)
Similarity	18	53.52 (24.02)
Dissimilarity	18	48.29 (19.05)
Integration	18	42.22 (20.62)
Inductive reasoning strategies	54	48.01 (18.67)

**Table 6:** Changes in the achievements of the control and the experimental group between the pre- and post-test.

Group	Pretest (%)		Posttest (%)		Change (%)	Pre- and Posttest	Effect Size (Cohen’s d)
	Mean	SD	Mean	SD		*t*-Test **	
Control (N = 55)	42.86	15.78	46.97	16.69	4.1	*t* = −3.336 *p* <.01	.25
Experimental (N = 67)	42.12	19.05	53.95	18.42	11.8	*t* = −9.057 *p* <.01	.63
*t*-test *	*t* = −.230 *p* = .82 n.s.		*t* = 2.173 *p* = .03			–	–

Note: SD: standard deviation. *: Independent samples *t*-test, **: Paired samples t-test.

**Table 7:** Changes in the achievements of the control and the experimental group in the inductive reasoning processes.

Inductive Reasoning Process	Control Group (%)		Change (%)	Exp. Group (%)		Change (%)	Corr. e.s. (Cohen’s d)
	Pre M. (SD)	Post M. (SD)		Pre M. (SD)	Post M. (SD)		
Generalization	48.1 (27.1)	54.9 (27.8)	6.9 *	49.8 (28.2)	64.5 (23.9)	14.8 **	.31
Discrimination	54.1 (22.5)	57.2 (23.8)	3.0	50.4 (24.0)	61.4 (24.2)	10.9 **	.32
Cross classification	32.7 (17.6)	33.5 (20.6)	0.8	35.2 (25.8)	37.1 (26.3)	2.0	.03
Recognizing relationships	42.0 (26.2)	48.1 (25.8)	6.1	40.3 (30.1)	59.0 (27.3)	18.7 **	.42
Differentiating relationships	37.0 (18.9)	39.8 (19.2)	2.8	33.5 (19.4)	48.3 (23.7)	14.8 **	.53
System construction	43.2 (24.4)	48.3 (25.3)	5.1	43.6 (23.9)	53.4 (24.4)	9.8 **	.20
Similarity	45.1 (22.1)	51.5 (22.9)	6.5 **	45.0 (25.3)	61.8 (22.5)	16.7 **	.41
Dissimilarity	45.6 (15.9)	48.5 (17.7)	2.9	42.0 (18.4)	54.8 (21.4)	12.9 **	.47
Integration	38.0 (19.9)	40.9 (18.5)	2.9	39.4 (20.2)	45.3 (20.1)	5.9 **	.13

Note: the level of significance is *p* < .01 **; the level of significance is *p* < .05 *. M.: mean, SD: standard deviation, Corr. e.s.: Corrected effect size.

## Data Availability

Not applicable.
